# A Method for Automated Detection of Chicken Coccidia in Vaccine Environments

**DOI:** 10.3390/vetsci12090812

**Published:** 2025-08-26

**Authors:** Ximing Li, Qianchao Wang, Lanqi Chen, Xinqiu Wang, Mengting Zhou, Ruiqing Lin, Yubin Guo

**Affiliations:** 1 College of Mathematics and Informatics, South China Agricultural University, Guangzhou 510642, China; liximing@scau.edu.cn (X.L.); qianchaowang@stu.scau.edu.cn (Q.W.); bailanking@stu.scau.edu.cn (L.C.); 2 Foshan Standard Bio-Tech Co., Ltd., Foshan 528138, China; wangxinqiu1987@126.com; 3 State Key Laboratory of Animal Nutrition and Feeding, Institute of Animal Science, Chinese Academy of Agricultural Sciences, Beijing 100193, China; zhoumengting@caas.cn; 4 College of Veterinary Medicine, South China Agricultural University, Guangzhou 510642, China; rqlin@scau.edu.cn

**Keywords:** chicken coccidiosis, chicken coccidia detection, vaccine quality, efficient multi-scale attention, multi-scale fusion, animal welfare

## Abstract

Vaccines play a crucial role in the prevention and control of chicken coccidiosis, effectively reducing economic losses in the poultry industry and significantly improving animal welfare. To ensure the production quality and immune effect of vaccines, accurate detection of chicken Coccidia oocysts in vaccine is essential. However, this task remains challenging due to the minute size of oocysts, variable spatial orientation, and morphological similarity among species. Therefore, we propose YOLO-Cocci, a chicken coccidia detection model based on YOLOv8n, designed to improve the detection accuracy of chicken coccidia oocysts in vaccine environments. Firstly, an efficient multi-scale attention (EMA) module was added to the backbone to enhance feature extraction and enable more precise focus on oocyst regions. Secondly, we developed the inception-style multi-scale fusion pyramid network (IMFPN) as an efficient neck. By integrating richer low-level features and applying convolutional kernels of varying sizes, IMFPN effectively preserves the features of small objects and enhances feature representation, thereby improving detection accuracy. Finally, we designed a lightweight feature-reconstructed and partially decoupled detection head (LFPD-Head), which enhances detection accuracy while reducing both model parameters and computational cost. The experimental results show that YOLO-Cocci achieves an mAP@0.5 of 89.6%, an increase of 6.5% over the baseline model, while reducing the number of parameters and computation by 14% and 12%, respectively. Notably, in the detection of Eimeria necatrix, mAP@0.5 increased by 14%. In order to verify the application effect of the improved detection algorithm, we developed client software that can realize automatic detection and visualize the detection results. This study will help improve the level of automated assessment of vaccine quality and thus promote the improvement of animal welfare.

## Simple Summary

This study proposed an automated detection method for chicken coccidia oocysts in vaccine environments. To accurately identify chicken coccidia oocysts in vaccines, we designed the YOLO-Cocci model, an optimized deep learning detection model. The model was improved in three key aspects, effectively enhancing the detection accuracy while reducing computational cost. In addition, we developed a user-friendly client software to automate and visualize the detection process. This method can help improve the automation level of vaccine quality assessment and thereby promote animal welfare in the poultry industry.

## 1. Introduction

The poultry industry is one of the most important sources of protein for the global population, but it faces many challenges, among which coccidiosis is one of the diseases that most significantly affects its production performance [1]. Chicken coccidiosis, an intestinal disease caused by protozoan parasites of the genus Eimeria, is one of the most serious diseases of livestock worldwide [2]. The disease is highly prevalent and widespread, severely affecting the feed conversion efficiency and growth of infected chickens, resulting in reduced production performance and increased mortality [3,4,5]. It causes severe economic losses worldwide, with an estimated annual economic loss of up to GBP 10.4 billion [6]. Consequently, preventing the occurrence of chicken coccidiosis is crucial to promote the continued growth of the poultry industry and protect economic income.

For decades, vaccines have played a crucial role in the prevention and control of chicken coccidiosis [7], providing a lasting solution to outbreaks of the disease. In order to ensure that the quality of vaccines produced meets standards, it is essential to accurately identify the species of chicken coccidia in vaccine samples and precisely count the number of each type of oocysts. Currently, the most common method for detecting chicken coccidia oocysts in vaccines relies on manual identification and counting by highly skilled technicians [8]. However, this method is not only labor intensive and time consuming, but also produces inconsistent results, making it difficult for it to meet the demands of modern vaccine production for high efficiency and accuracy.

In recent years, researchers have used molecular detection technology [9] to detect chicken coccidia. Although this method yields accurate results, its high cost and low feasibility make large-scale application difficult. At the same time, methods based on optical and electronic technologies [10,11,12] and digital image processing [13] have been gradually applied to chicken coccidia detection. Although these two types of methods have achieved certain success in identification and counting, they remain limited in effectively distinguishing impurity fragments that are similar in size to chicken coccidia oocysts, thereby affecting the detection accuracy.

With the rapid development of computer vision technology, chicken coccidia detection methods based on deep learning have shown great potential. Researchers have applied deep learning-based image detection algorithms to detect various microscopic organisms in microscope images, including pathogenic parasites [14], pathogenic fungi [15,16], other pathogens [17,18,19], and cells [20,21,22]. In general, deep learning-based image detection algorithms fall into two main categories: object detection methods and image segmentation methods.

In recent years, the YOLO series of models have gained wide attention in the field of object detection due to their excellent detection accuracy and fast processing speed. For example, Abdurahman et al. [23] used improved YOLOv3 and YOLOv4 models to detect malarial parasites in thick-blood-smear microscopy images, which outperformed the original versions of Faster R-CNN [24] and SSD [25] in terms of mean average precision, recall, precision, F1 score, and average IOU. Kumar et al. [26] used the YOLOv5 algorithm to detect and classify intestinal parasite eggs, achieving a mean average precision of about 97% on 5393 images, and the detection time for each sample was only 8.5 milliseconds. These object detection-based methods provide new solutions for chicken coccidia detection. However, current research on object detection for chicken coccidia remains limited and requires further exploration and optimization.

The basic principle of image segmentation technology is to extract the boundaries of targets through pixel-level division, thereby achieving accurate recognition. Smith et al. [27] used a segmentation model combining U-NET and StarDist, along with a CNN-based classification model, to achieve automatic recognition and counting of three Eimeria oocyst types in fecal samples. Kellogg et al. [28] employed the Mask R-CNN [29] segmentation model to detect three Eimeria species in infected chickens and determine the sporulation state of their oocysts. However, these methods have certain limitations. Specifically, such models often have a high number of parameters and computational costs, and there is still room for improvement in accuracy. In addition, existing studies have not yet conducted detection of morphologically similar chicken coccidia oocysts.

Despite progress in the deep learning-based detection method for chicken coccidia, many challenges and concerns remain. Firstly, because chicken coccidia datasets from vaccine environments are difficult to obtain, most research currently focuses on the detection of chicken coccidia oocysts in fecal samples, with relatively few studies conducted in vaccine environments. Secondly, chicken coccidia oocysts in microscope images are usually small, exhibit variable spatial orientation, and show morphological similarity among species, all of which increase the difficulty of detection. Finally, the accuracy of existing object detection algorithms in detecting multi-category chicken coccidia still needs improvement, especially in identifying oocysts with similar morphology but belonging to different species (such as E. necatrix and E. tenella), which remains a significant challenge. To this end, this paper proposes an improved object detection model, YOLO-Cocci (where “Cocci” is the abbreviation for Coccidia), based on the YOLOv8n architecture. The proposed model aims to enhance the accuracy of chicken coccidia detection in vaccine environments and achieve accurate identification of four Eimeria species (E. acervulina, E. necatrix, E. maxima, E. tenella) and their spore formation state. We also developed a user-friendly client software for efficient automated detection in real-world vaccine scenarios.

The main contributions of this study are as follows:

1. We constructed a chicken coccidia dataset suitable for vaccine environments. The dataset includes four Eimeria species, and contains both sporulated and non-sporulated morphologies of each species, providing rich and diverse samples for the chicken coccidia detection task.
2. The YOLO-Cocci model significantly improves the detection accuracy of chicken coccidia through three key improvements. First, an efficient multi-scale attention (EMA) module is integrated into the backbone to enhance the feature extraction of chicken coccidia oocysts. Second, the original neck is replaced with an inception-style multi-scale fusion pyramid network (IMFPN), which utilizes multi-scale feature fusion and parallel deep convolution to better retain critical features and enhance feature representation ability. Finally, a lightweight feature-reconstructed and partially decoupled detection head (LFPD-Head) is employed to further improve accuracy and optimize performance.
3. The results of comparative experiments show that the YOLO-Cocci model outperforms other object detection models on the chicken coccidia dataset. Ablation studies further verifies its advantages in detecting morphologically similar oocysts. To improve user experience, a user-friendly client was developed for automatic detection and visualization of the YOLO-Cocci results. This study provides essential technical support for detecting chicken coccidia in vaccine environments.

The rest of this paper is organized as follows: Section 2 describes the construction process of the chicken coccidia dataset in detail and introduces the YOLO-Cocci detection model. Section 3 provides the experimental results and an in-depth analysis of them, and introduces the implementation of the automated detection system. Section 4 points out the limitations of this study and discusses future research directions. Section 5 summarizes the main work of this study.

## 2. Materials and Methods

**Figure 1:**
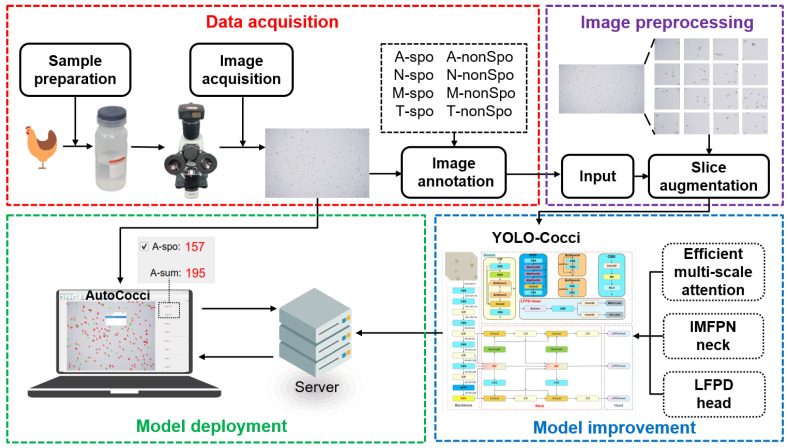
The overall research route of this study.

In order to achieve high-precision automated detection of chicken coccidia oocysts, this study proposes the YOLO-Cocci model and deploys it on the server. Users can perform automated detection by invoking the model. The overall research route is illustrated in Figure 1.

### 2.1. Data Acquisition

The chicken coccidia dataset used in this study was gathered from December 2023 to April 2024 in the vaccine production room of standard Bio-Tech Co., Ltd. in Sanshui District, Foshan City.

#### 2.1.1. Chicken Eimeria Preparation

To prepare the four chicken coccidia sample fluids, non-sporulated oocysts were first extracted from chicken manure using the saturated saline flotation method. Subsequently, the oocysts were cultured at 28 °C in a constant-temperature shaker and 150 rpm for 22 to 30 h to promote sporulation. Next, the sporulated oocysts were inoculated into chicks, and the chicken manure was collected 5 to 10 days after inoculation. The collected feces were placed in a container, an appropriate amount of clean water was added, and the mixture was stirred thoroughly to dissolve the feces. It was then filtered through 60-mesh, 100-mesh, and 200-mesh sieves in sequence to collect the filtrate. The filtrate was then centrifuged at 1800 rpm for 2 min, and the supernatant was discarded. After all the precipitate was collected, an appropriate amount of saturated saline was added to resuspend it, and it was centrifuged at 2800 rpm for 3 min to collect the supernatant. The precipitate was then resuspended in saturated saline and observed under a microscope. If a large number of oocysts were visible in the resuspension, the supernatant was collected by centrifugation; if fewer than 100 oocysts were visible in each field of view, no further collection was performed. The collected supernatant was diluted with five times its volume of clean water and centrifuged at 2800 rpm for 3 min to collect the precipitate, which was the crude extract of the oocysts. Subsequently, 1% chloramine T was added to the crude extract to promote sporulation. Finally, four chicken coccidia sample solutions were prepared, covering four species: E. acervulina, E. necatrix, E. maxima, and E. tenella. Each sample solution contained both sporulated and non-sporulated forms of the respective species.

#### 2.1.2. Image Acquisition

**Figure 2:**
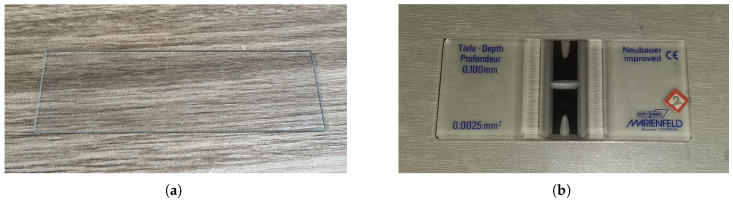
Two types of carriers for sample solutions: (**a**) Glass slide. (**b**) Imported blood-counting plates.

**Figure 3:**
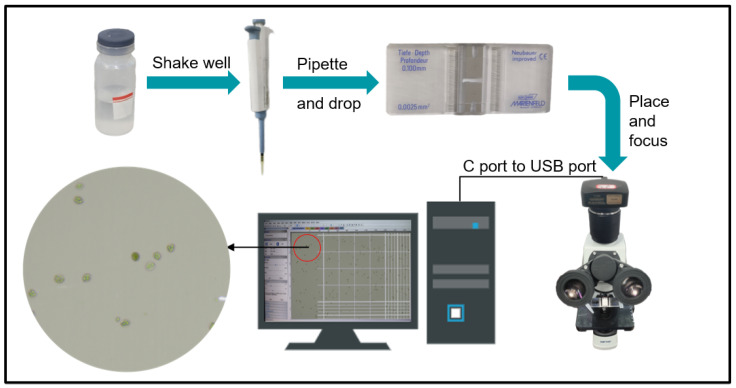
Chicken coccidia image acquisition process.

This study used a camera equipped with a 20-megapixel sensor (model: E3ISPM20000KPA) mounted on a biological microscope for image acquisition. The camera features a 1-inch sensor (size: 13.06 × 8.76 mm) and utilizes its dedicated software to complete image capture, ensuring high-quality image data acquisition. The microscope used is a Shunyu EX20, with a magnification of 100× during image acquisition. The sample carrier includes a glass slide and an imported blood cell-counting plate (model: Bright Line 0650030), as shown in Figure 2a,b. The image acquisition process is as follows: First, a pipette is used to absorb the shaken chicken coccidia sample, which is then dropped onto the carrier and covered with a cover glass. Next, the carrier is placed on the microscope stage for focusing. Finally, the image is captured using the ImageView.exe software. When using an imported blood cell-counting plate as the carrier, the specific image acquisition process is shown in Figure 3. To ensure image clarity, the parameters for each shot were kept consistent, including automatic white balance, automatic exposure, and other default settings. A total of 420 JPG images with a resolution of 5440 × 3648 pixels were collected. Specifically, there were 100 images containing E. acervulina oocysts, 107 images containing E. necatrix oocysts, 111 images containing E. maxima oocysts, and 102 images containing E. tenella oocysts. For details, see Table 1.

#### 2.1.3. Image Annotation

**Figure 4:**
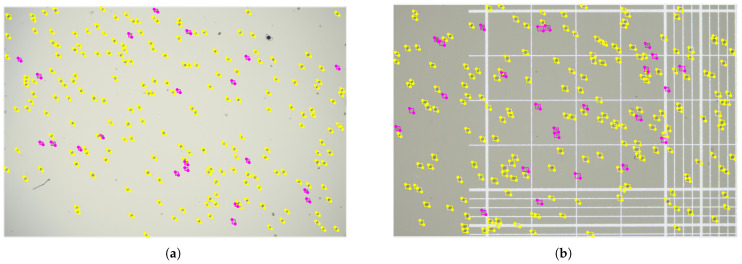
Image annotation for two carrier types: (**a**) Image labeling on glass slide. (**b**) Image labeling on imported blood-counting plate.

**Figure 5:**
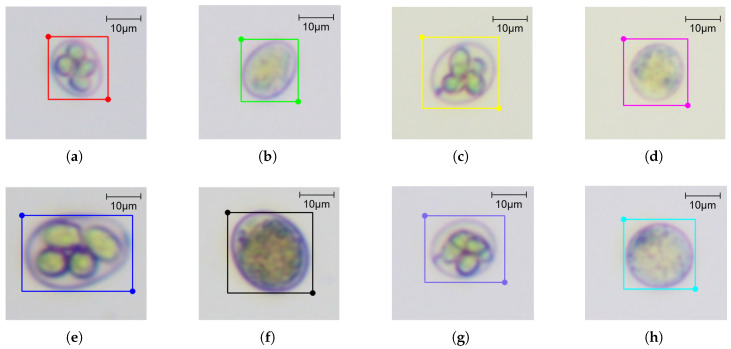
Examples of oocysts corresponding to each label type: (**a**) sporulation of E. acervulina oocysts; (**b**) non-sporulation of E. acervulina oocysts; (**c**) sporulation of E. necatrix oocysts; (**d**) non-sporulation of E. necatrix oocysts; (**e**) sporulation of E. maxima oocysts; (**f**) non-sporulation of E. maxima oocysts; (**g**) sporulation of E. tenella oocysts; (**h**) non-sporulation of E. tenella oocysts.

The labelme tool was used (https://github.com/wkentaro/labelme) (accessed on 24 August 2025) to annotate the chicken coccidia oocysts in the image with rectangles (Figure 4a,b). A total of 8 annotated categories were included, covering four different species of chicken coccidia and the sporulated and non-sporulated forms of each species. The specific labels are as follows: A-spo, A-nonSpo, N-spo, N-nonSpo, M-spo, M-nonSpo, T-spo, and T-nonSpo, which, respectively, represent sporulation of E. acervulina oocysts, non-sporulation of E. acervulina oocysts, sporulation of E. necatrix oocysts, non-sporulation of E. necatrix oocysts, sporulation of E. maxima oocysts, non-sporulation of E. maxima oocysts, sporulation of E. tenella oocysts, and non-sporulation of E. tenella oocysts, as shown in Figure 5a–h. Among these, sporulated oocysts are identified by checking whether the oocyst contains four sporangia; the characteristic of non-sporulated oocysts is that a sporophyte occupies most of the oocyst. After the annotation was completed, based on the four chicken coccidia species and two carrier types in the dataset, we randomly divided the data of each carrier type in each category into training, validation, and test sets in a 6:2:2 ratio.

### 2.2. Image Preprocessing

**Figure 6:**
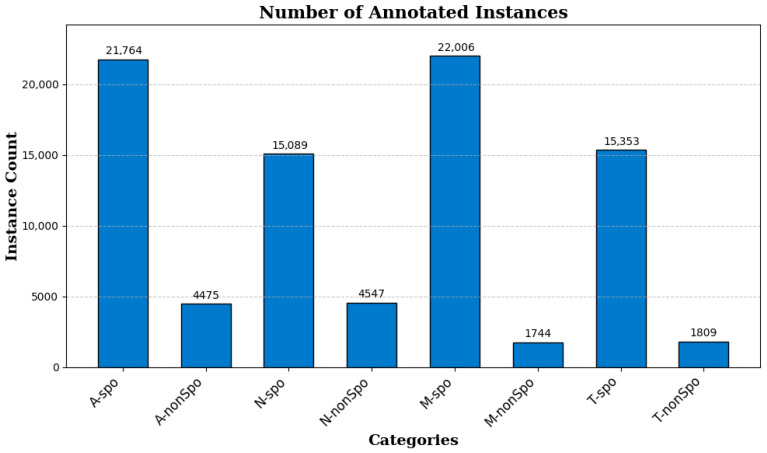
Number of annotated instances per category label.

Since a single chicken coccidia oocyst occupies a small proportion of the pixels in an image, directly using the original image as input may make it difficult to capture the details of the oocyst, and the computational overhead would be high. To address this, we adopted a slicing data augmentation strategy. Specifically, the length and width of the image are first padded to the nearest integer multiple of 640, and then the padded image is divided into multiple non-overlapping 640 × 640 pixel regions. We applied this slicing strategy during the training and validation phases. To simulate the real-world detection scenario of chicken coccidia in vaccine environments, the images in the test phase (a total of 83 images) retained their original resolution (5440 × 3648). Using this method, the training set was expanded to 12,804 images and the validation set to 4272 images. The number of annotated instances corresponding to each category label is shown in Figure 6.

### 2.3. Baseline Model

**Figure 7:**
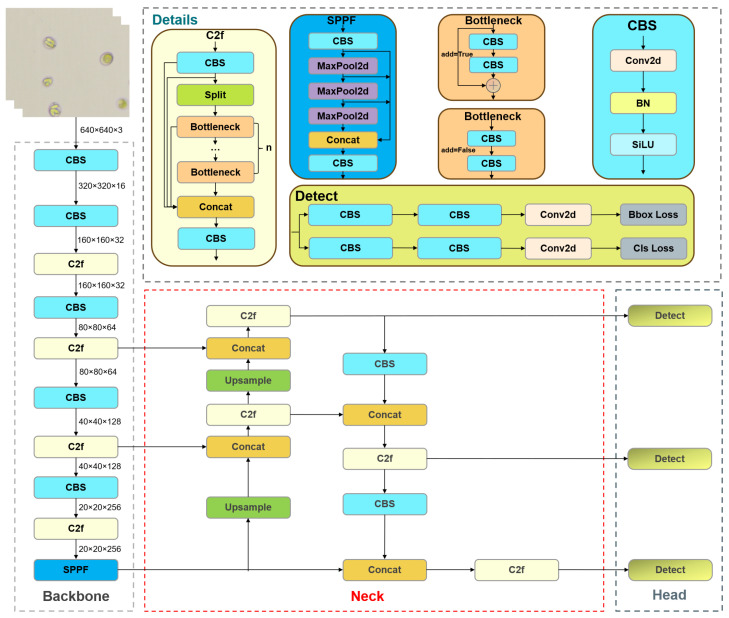
YOLOv8 model structure.

YOLOv8 is the next-generation object detection model proposed by the Ultralytics team based on YOLOv5. Compared with YOLOv5, YOLOv8 utilizes a more advanced backbone network and neck architecture. For instance, YOLOv8 substitutes the C3 module in its backbone and neck structures with the C2f module, thereby improving the feature extraction capability and object detection performance. Additionally, YOLOv8 introduces an anchor-free decoupling head, which significantly improves detection accuracy and efficiency compared with the anchor-coupled head in YOLOv5. YOLOv8 also incorporates various online data enhancement techniques, including Mosaic data augmentation [30], Mixup data augmentation [31], HSV channel transformation, image scaling, and horizontal or vertical flipping. After several updates, the current version of YOLOv8 has been updated to 8.2.103. YOLOv8 provides models of different scales, including n, s, m, l, and x, to meet various application requirements. This study used the Ultralytics version 8.2.50 (https://github.com/ultralytics/ultralytics/tree/v8.2.50 (accessed on 24 August 2025) ) of YOLOv8n as the baseline model, and its structure is shown in Figure 7.

### 2.4. YOLO-Cocci Model

**Figure 8:**
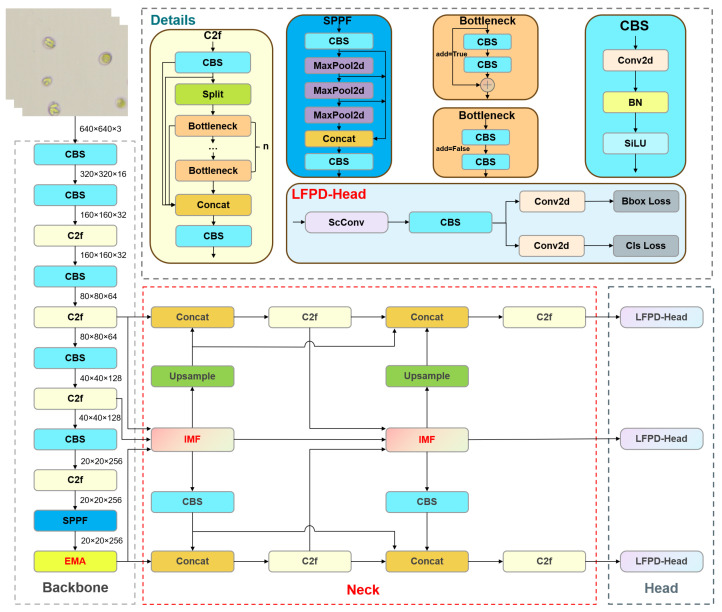
YOLO-Cocci model structure. The red dashed part of the figure represents the improved neck structure proposed in this paper.

The YOLO-Cocci model mainly consists of backbone, neck, and detection head. The overall structure is shown in Figure 8. Taking into account the specific features of the chicken coccidia dataset, the model is enhanced across three aspects: (1) An EMA [32] module is added at the end of the backbone network. Through multi-scale feature fusion and cross-dimensional interaction, the model’s attention to chicken coccidia oocysts is enhanced, thereby reducing false detections; (2) given the small size of chicken coccidia oocysts in microscope images, their reliance on details in low-level features, and their variable spatial orientation, we introduced the IMFPN structure. IMFPN fuses features of different scales through feature concatenation and uses multi-scale deep convolution kernels to extract multi-scale information across receptive fields, which effectively retains more key features of chicken coccidia and enhances feature representation ability, thereby improving detection accuracy; (3) we introduced LFPD-Head to reduce feature redundancy and enhance the expression ability of low-level features, improving detection accuracy while effectively reducing the number of model parameters and computational cost.

#### 2.4.1. The EMA Module

**Figure 9:**
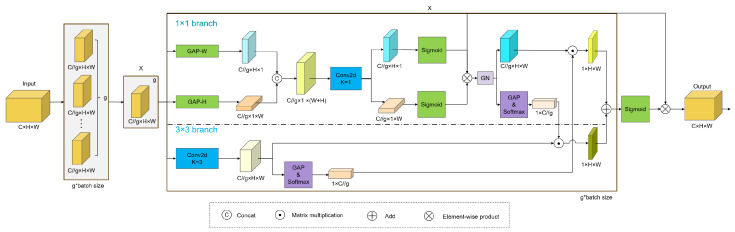
EMA module.

With its flexible structural characteristics, the attention mechanism can both enhance the learning of discriminative features and be easily embedded in the model backbone network, thereby improving the detection performance of the neural network [33,34]. Additionally, different categories of chicken coccidia often exhibit diversity in morphology and scale in images. To this end, we introduced the EMA attention module at the end of the backbone network, which facilitates efficient multi-scale feature fusion and cross-dimensional interaction with low parameter overhead, captures pixel-level pairwise relationships, and constructs both long-range and short-range dependencies. This enhances the model’s ability to understand and capture chicken coccidia features, effectively reducing false detections and significantly improving detection accuracy. Structurally, the EMA module consists of two branches: one using a 1 × 1 convolution kernel and the other using a 3 × 3 convolution kernel, referred to as the 1 × 1 branch and 3 × 3 branch, respectively. Its overall structure is shown in Figure 9.

For any given input feature map $X\in R^{C\times H\times W}$, the EMA module divides *X* into *G* groups of sub-feature maps along the channel dimension to learn different semantic information. The representation of each group of sub-feature maps is $X=\left[X_{0},X_{i},\ldots ,X_{G-1}\right],X_{i}\in R^{C//G\times H\times W}$, where *C* represents the number of channels of the input feature map, and *H* and *W* represent the height and width of the input feature map, respectively.

The EMA module uses three parallel paths to extract the attention weight descriptors for each group of sub-feature maps: two within the 1 × 1 branch and one in the 3 × 3 branch. In the 1 × 1 branch, two one-dimensional global average pooling (GAP) operations, called GAP-W and GAP-H, encode channels along the horizontal and vertical directions to generate direction-aware features $F_{c}^{W}\in R^{C//G\times H\times 1}$ and $F_{c}^{H}\in R^{C//G\times 1\times W}$, which are calculated as follows:

(1) $$F_{c}^{W}\left(w\right)=\frac{1}{H}\sum_{0\leq i<H}x_{c}\left(i,w\right)$$

(2) $$F_{c}^{H}\left(h\right)=\frac{1}{W}\sum_{0\leq j<W}x_{c}\left(h,j\right)$$

Next, $F_{c}^{W}$ and $F_{c}^{H}$ are concatenated along the image height direction and linearly transformed through a shared 1 × 1 convolution layer. Its output is decomposed into two vectors, and attention weights are generated through two nonlinear Sigmoid functions. Subsequently, the two channel attention maps in each group are aggregated through matrix multiplication, and finally group normalization (GN) [35] is applied to the result. Meanwhile, the 3 × 3 branch uses 3 × 3 convolution to capture local cross-channel interactions, thereby expanding the feature space and enhancing multi-scale feature representation.

The output of GN is processed by 2D GAP and Softmax nonlinear activation. Then, the activation result is matrix-multiplied with the sub-feature map obtained by 3 × 3 convolution to generate the first spatial attention map, which integrates spatial information at different scales. The 2D GAP operation is formulated as

(3) $$z_{c}=\frac{1}{H\times W}\sum_{i=1}^{H}\sum_{j=1}^{W}x_{c}\left(i,j\right)$$

where $x_{c}\left(i,j\right)$ is the input value of the *c*-th channel at the spatial position (i,j), and $z_{c}$ represents the pooled output of the *c*-th channel. Additionally, 2D GAP is also used to encode the global spatial information in the 3 × 3 branch, followed by nonlinear activation using the Softmax function. The Softmax activation result is matrix-multiplied with the 1 × 1 branch’s GN output to generate a second spatial attention map retaining accurate spatial position details. Then, the two spatial attention maps from each group are summed, followed by a Sigmoid activation to produce the final attention weight. This attention weight captures pixel-level pairwise relationships and strengthens the global context of all pixels. Finally, by multiplying this attention weight element-wise by the original input feature map, an output feature map that better highlights the region of interest is generated.

#### 2.4.2. The IMFPN

**Figure 10:**
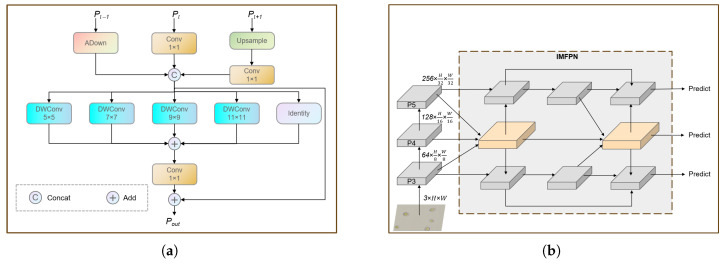
(**a**) IMF module. (**b**) Architecture of the IMFPN.

The neck network of YOLOv8 adopts the PAFPN [36] structure, adding a bottom-up path based on the FPN [37] to compensate for the lack of low-level feature details in the high-level features of FPN. However, this design may result in information loss or degradation of low-level features during propagation and interaction. This problem is particularly critical for the detection of chicken coccidia oocysts, as oocysts are typically small and their recognition relies on the detailed information in low-level features. Furthermore, oocysts exhibit multidimensional variations in size and spatial orientation, further increasing the challenge of detection. To solve the above problems, we proposed an Inception-style Multi-scale Fusion (IMF) module (Figure 10a) to enhance the feature representation ability of the neck network, thereby improving the model’s adaptability to chicken coccidia oocysts of various sizes and orientations. Based on this, we employed this module to reconstruct the neck network, forming IMFPN (Figure 10b), which strengthens the extraction of key low-level features and enhances its ability to detect chicken coccidia. This design not only optimizes the feature transfer mechanism, but also effectively improves the detection accuracy.

The IMF module fuses feature map inputs from three different scales and extracts multi-scale texture features across different receptive fields through a series of parallel deep convolutions to enhance the model’s feature representation capabilities. Specifically, we denote the three different scale features as $\left\{P_{l-1},P_{l},P_{l+1}\right\},P_{l}\in R^{C_{l}\times \frac{H}{2^{l}}\times \frac{W}{2^{l}}}$. In the IMF module, these features are first converted to feature maps with the same spatial scale as $P_{l}$, and their number of channels is compressed to $\frac{C_{l}}{2}$. Subsequently, the three feature maps are concatenated channel-wise to produce the fused feature $f\in R^{\frac{3}{2}\times C_{l}\times \frac{H}{2^{l}}\times \frac{W}{2^{l}}}$. This conversion process can be expressed as

(4) $$f=[\mathrm{ADn}\left(P_{l-1}\right),\mathrm{Cov}\left(P_{l}\right),\mathrm{Cov}\left(\mathrm{Upe}\left(P_{l+1}\right)\right)]$$

where [·, ·, ] denotes the concatenation operation along the channel dimension, ADn refers to applying the ADown module for downsampling, Cov means applying the convolution module for channel compression, and Upe means applying upsampling. In this study, l=4. Using the ADown module for downsampling helps to preserve as much detailed information as possible in the low-level features.

Next, *f* is fed into a set of parallel depthwise convolutions to capture contextual information across multiple scales. The output feature maps from these different receptive fields are added together, and a 1×1 convolution is used to fuse the local features with the contextual features. Finally, the convolution result is added to *f* to obtain the final output feature map $P_{\mathrm{out}}$. The above process can be expressed as

(5) $$Z=\sum_{m=1}^{4}{\mathrm{DWConv}}_{k^{\left(m\right)}\;\times \;k^{\left(m\right)}}\left(f\right),\;m=1,\ldots ,4$$

(6) $$P_{\mathrm{out}}=f+{\mathrm{Conv}}_{1\times 1}\left[f+Z\right]$$

The 1×1 convolution is used as a channel fusion mechanism to integrate features from different receptive field sizes. This allows the IMF module to effectively capture the extensive contextual information of chicken coccidia oocysts and enhances the model’s ability to perceive chicken coccidia oocysts at varying sizes and orientations, thereby significantly improving accuracy.

#### 2.4.3. The LFPD-Head Module

**Figure 11:**
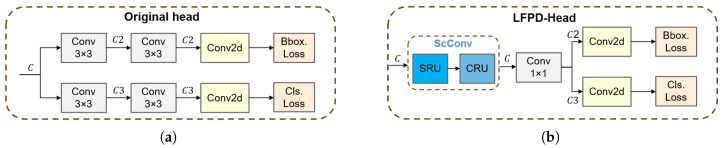
(**a**) The original head. (**b**) The architecture of LFPD-Head. In both designs, the channel dimensions C2 and C3 satisfy C2, C3 ≤ C.

The detection head of YOLOv8 (Figure 11a) adopts a decoupled design, where the regression head and the classification head extract target feature information through two convolutional layers, with channel dimension reduction occurring in the first convolutional layer. Although this design effectively reduces the computational complexity and parameter scale of the model, premature dimension reduction may result in insufficient representation of low-level features, which could affect detection performance. To this end, this study introduces the LFPD-Head (Figure 11b), which adopts a partially decoupled structure and incorporates spatial and channel reconstruction convolution (ScConv) [38] to reduce feature redundancy and enhance feature representation capabilities. Additionally, channel dimension reduction is postponed until the second convolutional layer, enabling the model to learn richer low-level features, thereby improving detection accuracy.

**Figure 12:**
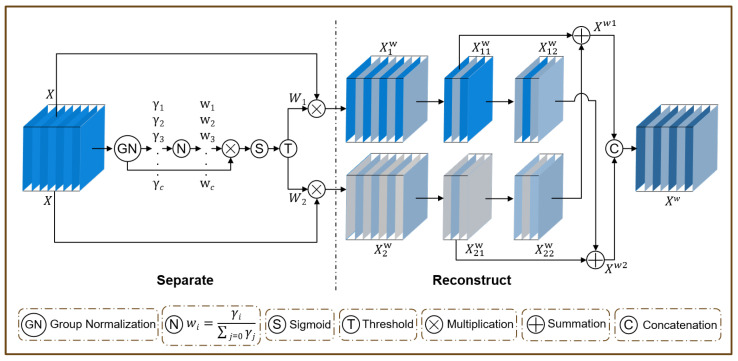
Design of spatial reconstruction unit.

**Figure 13:**
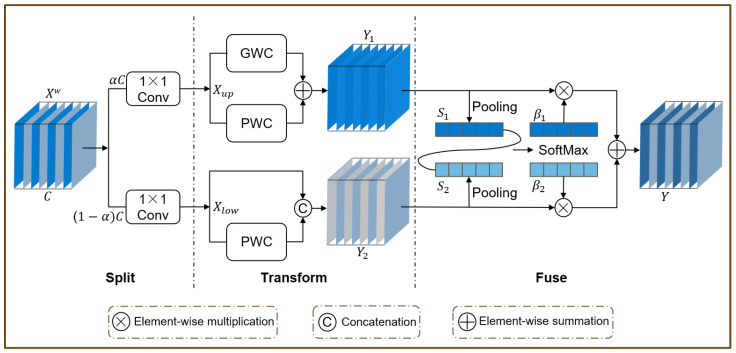
Design of the channel reconstruction unit.

ScConv consists of two units, namely, the spatial reconstruction unit (Figure 12) and the channel reconstruction unit (Figure 13), which are placed in sequence. Specifically, for the input feature $X\in R^{C\times H\times W}$, the spatial-refined $X^{w}$ feature is first obtained through the SRU operation, and then the channel-refined feature *Y* is obtained using the CRU operation.

In the SRU operation, the scaling factor $\gamma =[\gamma_{1},\gamma_{2},\gamma_{3},\ldots ,\gamma_{c}]$ is first obtained through GN; γ is used to evaluate the information content of different feature maps. Richer spatial information reflects more changes in spatial pixels, resulting in a larger γ. Then, γ is normalized to obtain the weight coefficient $w=[w_{1},w_{2},w_{3},\ldots ,w_{c}]$. The normalization process can be expressed as

(7) $$W_{\gamma }=\left\{w_{i}\right\}=\frac{\gamma_{i}}{\sum_{j=1}^{C}\gamma_{j}},i,j=1,2,\ldots ,C$$

Then, the weight values of the feature maps re-weighted by $W_{\gamma }$ are mapped to (0, 1) through the Sigmoid function and gated. The weights with a mapping value greater than or equal to 0.5 are set to 1 to obtain the informative weight $W_{1}$; the weights with a value less than 0.5 are set to 0 to obtain the non-informative weight $W_{2}$. The entire process of obtaining W can be expressed as

(8) $$W=\mathrm{Gate}\left(\sigma \left(W\gamma \left(\mathrm{GN}(X)\right)\right)\right)$$

Next, the input feature *X* is multiplied by $W_{1}$ and $W_{2}$ to obtain two weighted features; with the feature $X_{1}^{w}$ having more information and the feature $X_{2}^{w}$ less information. Then, $X_{1}^{w}$ is equally divided into two parts according to the channel dimension to obtain $X_{11}^{w}$ and $X_{12}^{w}$. Similarly, $X_{2}^{w}$ is divided into $X_{21}^{w}$ and $X_{22}^{w}$. Next, a cross-reconstruction operation is used to fully combine the two weighted different information features and strengthen the information flow between them. The cross-reconstruction operation can be expressed as

(9) $$X_{11}^{w}⊕X_{22}^{w}=X^{w1}$$

(10) $$X_{21}^{w}⊕X_{12}^{w}=X^{w2}$$

where ⊕ is element-wise summation. Finally, the cross-reconstructed features $X^{w1}$ and $X^{w2}$ are concatenated to obtain the spatial-refined feature map $X^{w}$.

The CRU operation adopts a split, transform, and fuse strategy. The split strategy mainly includes two steps: splitting and squeezing. Specifically, for a given spatial-refined feature $X^{w}\in R^{c\times h\times w}$, $X^{w}$ is first split into two feature maps, αc×h×w and (1−α)c×h×w, along the channel dimension ($\alpha =\frac{1}{2}$ in the experiment). Subsequently, a 1×1 convolution is applied to squeeze the number of channels of the feature map to half of the original, improving computational efficiency. After squeezing, the upper feature map $X_{up}$ and the lower feature map $X_{low}$ are obtained. In the transform stage, $X_{up}$ serves as the input to the upper transformation stage, acting as a rich feature extractor. The high-level representative information is extracted using 3×3 group-wise convolution (GWC) and 1×1 point-wise convolution (PWC), thereby replacing the expensive standard 3×3 convolution operation and reducing the computational cost. The output is then aggregated to form a merged representative feature map $Y_{1}$. The upper transformation stage can be formulated as

(11) $$Y_{1}=M^{G}X_{up}+M^{P_{1}}X_{up}$$

where $M^{G}\in R^{\frac{\alpha c}{2g}\times 3\times 3\times c}$, $M^{P_{1}}\in R^{\frac{\alpha c}{2}\times 1\times 1\times c}$ are the learnable weight matrices for GWC and PWC; and $X_{up}\in R^{\frac{\alpha c}{2}\times h\times w}$ and $Y_{1}\in R^{c\times h\times w}$ are the input and output feature maps of the upper layer, respectively. $X_{low}$ is fed into the lower transformation stage, where a cheap 1×1 PWC operation is used to generate feature maps with shallow hidden details as a complement to the rich feature extractor. Finally, the generated features are concatenated with the $X_{low}$ features to form the output of the next level $Y_{2}$, as shown below:

(12) $$Y_{2}=M^{P_{2}}X_{\mathrm{low}\;}\cup X_{\mathrm{low}\;}$$

where $M^{P_{2}}\in R^{\frac{(1-\alpha )c}{2}\times 1\times 1\times \left(1-\frac{1-\alpha }{2}\right)c}$ is the learnable weight matrix of PWC, ∪ is the cascade operation, and $X_{low}\in R^{\frac{(1-\alpha )c}{2}\times h\times w}$ and $Y_{2}\in R^{c\times h\times w}$ are the input and output feature maps of the lower layer, respectively. After the transformation is completed, the simplified SKNet method [39] is used to adaptively merge the output features $Y_{1}$ and $Y_{2}$ of the up and down transformation stages, as shown in the fuse section in Figure 13. First, global average pooling is applied to collect global spatial information with channel statistics $S_{1},S_{2}\in R^{c\times 1\times 1}$. Then, the upper and lower global channel descriptors $S_{1}$, $S_{2}$ are stacked together, and the channel soft attention operation is used to generate the feature importance vector $\beta_{1},\beta_{2}\in R^{c}$, as shown below:

(13) $$\beta_{1}=\frac{e^{s_{1}}}{e^{s_{1}}+e^{s_{2}}},\;\beta_{2}=\frac{e^{s_{2}}}{e^{s_{1}}+e^{s_{2}}},\;\beta_{1}+\beta_{2}=1$$

Finally, guided by the feature importance vectors $\beta_{1}$, $\beta_{2}$, the upper features $Y_{1}$ and the lower features $Y_{2}$ are merged in a channel-wise manner to obtain the channel-refined features *Y*, as shown below:

(14) $$Y=\beta_{1}Y_{1}+\beta_{2}Y_{2}$$

The improved detection head not only learns richer feature representations of chicken coccidia but also lowers model complexity and computational demands.

## 3. Results

### 3.1. Experimental Setup

The experiment was conducted on a server equipped with an AMD R9 7900X 5.6 GHz CPU, 64 GB RAM, and an NVIDIA RTX 4090D GPU, with an operating system of Ubuntu 22.04. The deep learning framework used was PyTorch 2.4.1, the CUDA and CUDNN versions were 12.6 and 9.0, respectively, the Python version was 3.10, and the integrated development environment was VSCode. In this experiment, the training parameters were set as follows: epochs was 550, batch size was 128, and image size was 640 × 640. The optimizer used was Adamax, with a momentum of 0.937 and a weight decay of 0.0005. The initial learning rate was set to 0.01, and a warm-up strategy was used to automatically adjust the learning rate in the first three epochs. In order to avoid overfitting, an early-stopping strategy was used during training. In the test phase, IoU and confidence were set to 0.5, and max_det was set to 400 to ensure that all chicken coccidia oocysts in the image could be detected. In addition, a variety of online data enhancement strategies were used during the training process, including mosaic enhancement, random horizontal and vertical flipping, HSV channel transformation, etc.

### 3.2. Evaluation Metrics

This study uses precision (P), recall (R), mean average precision (mAP), parameters (Params), and GFLOPs as evaluation indicators to evaluate the overall performance of the model. P, R, and mAP are calculated as follows:

(15) $$P=\frac{TP}{TP+FP}\times 100\%$$

(16) $$R=\frac{TP}{TP+FN}\times 100\%$$

(17) $$AP=\int_{0}^{1}P\left(r\right)d_{r}$$

(18) $$mAP=\frac{1}{C}\sum_{i=1}^{C}AP_{i}\times 100\%$$

Among these, *TP*, *TN*, *FP*, and *FN* represent true positives, true negatives, false positives, and false negatives, respectively. In the ablation experiments, we use the average precision when the IoU threshold is 0.5 as the reference metric.

### 3.3. Comparison Experiment

To verify the superiority of the proposed model, we conducted comparative experiments with a series of state-of-the-art YOLO models (YOLOv5, YOLOv9 [40], YOLOv10 [41], YOLOv11 [42]) and classic two-stage models (RetinaNet [43], Faster R-CNN, Mask R-CNN), involving seven models. All experiments were performed under the same dataset and experimental conditions, with each model trained until convergence. Detailed comparative experiment results are summarized in Table 2.

As presented in Table 2, the YOLO-Cocci model outperforms the comparison models in terms of mAP@0.5, mAP@0.5:0.95, and P. Although the YOLO-Cocci model achieved the second-highest score in the R indicator (86.0%), its computational cost and number of parameters are lower than those of YOLOv11n (86.4%). Compared with the YOLOv11n model, although the YOLO-Cocci model has more parameters and higher computational complexity, its scores in mAP@0.5, mAP@0.5:0.95, P, and R are improved by 1.3%, 0.7%, 0.1%, and 2.0%, respectively. Compared with the YOLOv5n model, although its frames per second (FPS) are lower, its detection accuracy is higher. Additionally, compared with the two-stage models, the YOLO-Cocci model surpasses them in both overall scale and accuracy. In general, the proposed model has an advantage in overall performance.

### 3.4. Ablation Study

#### 3.4.1. Ablation Experiments for Multi-Scale Kernel Design of IMF Module

To evaluate the effect of different multi-scale kernels in the IMF module in deep convolution, we conducted ablation experiments on the multi-scale kernel design, and the results are presented in Table 3. The experiments show that using only 3 × 3 kernels results in poor performance due to the limited extraction of local context information. When adopting the multi-scale kernel structure, the model achieves optimal performance with kernel sizes ranging from 5 × 5 to 11 × 11 and a stride of 2. When the kernel size is further increased, the performance begins to degrade. Additionally, attempts to increase the number of kernels or adjust the stride also lead to performance degradation. Based on these experimental observations, we selected the configuration of kernel sizes (5, 7, 9, 11) for the IMF module.

#### 3.4.2. Overall Ablation Experiments of the Improved YOLOv8 Model

In this section, to verify the effectiveness of the improved model, we conducted ablation experiments on three improvement strategies: (1) introducing the EMA module; (2) redesigning the neck network with IMFPN; (3) introducing the LFPD-Head module. The results of the experiments are presented in Table 4. As shown in Table 4, when the baseline network introduces the IMFPN, the model performance improves significantly. With almost no increase in the number of parameters, mAP@0.5 and mAP@0.5:0.95 are increased by 3.7% and 2.8%, respectively, and the accuracy of chicken coccidia detection in each category also improves, with the exception of the M-spo category. When both the EMA and IMFPN modules are used together, compared with other pairwise combination models, mAP@0.5 and mAP@0.5:0.95 achieve the best level, 89.1% and 67.1%, respectively, and the average precision of M-spo is also the highest. This is because IMFPN can directly fuse feature maps from non-adjacent layers, enhancing their representation capabilities. It retains the important features of the chicken coccidia oocyst while also extracting contextual information across different scales, thereby improving the model’s feature representation ability. Meanwhile, the EMA module focuses on more relevant features at the pixel level and suppresses less useful ones, thereby improving the model’s detection accuracy.

Although EMA+IMFPN and EMA+LFPD-Head achieve comparable mAP@0.5, EMA+IMFPN demonstrates superior performance at mAP@0.5:0.95. This suggests that IMFPN effectively captures multi-scale spatial context, which is critical for accurately detecting chicken coccidia with varying spatial orientations and scales. In contrast, the LFPD-Head performs feature reconstruction with lower computational effort and lacks sufficient spatial context enhancement, which may lead to lower detection accuracy.

**Figure 14:**
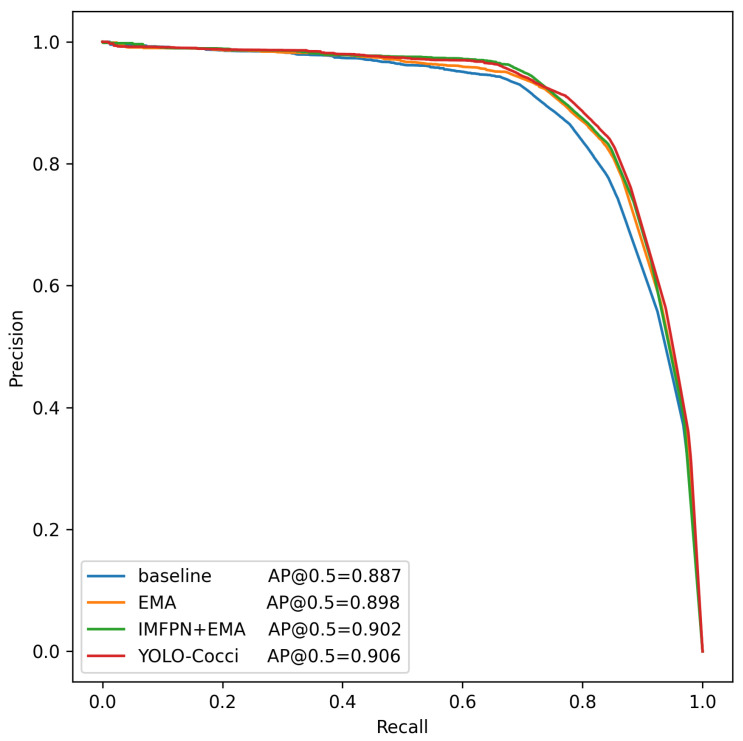
P-R curves of different improved models on the validation set.

When all three modules are integrated into the baseline model, the mAP@0.5 and mAP@0.5:0.95 of the YOLO-Cocci model improve by 6.5% and 5.0%, respectively, while reducing the parameter count and computational cost. Additionally, compared with the baseline model, YOLO-Cocci shows improved average precision in the A-spo, A-nonSpo, N-spo, N-nonSpo, M-spo, M-nonSpo, T-spo, and T-nonSpo categories by 7.0%, 5.7%, 14%, 12.5%, 0.3%, 0.2%, 3.6%, and 8.3%, respectively. Figure 14 further demonstrates the YOLO-Cocci model’s advantages in overall recall and precision. These results confirm that the improved modules significantly enhance chicken coccidia detection. Particularly in the N-spo category, where the model achieved the most significant improvement in average precision, this highlights YOLO-Cocci’s strong ability to differentiate morphologically similar chicken coccidia, further confirming its superiority in this detection task.

### 3.5. EMA Visualization

**Figure 15:**
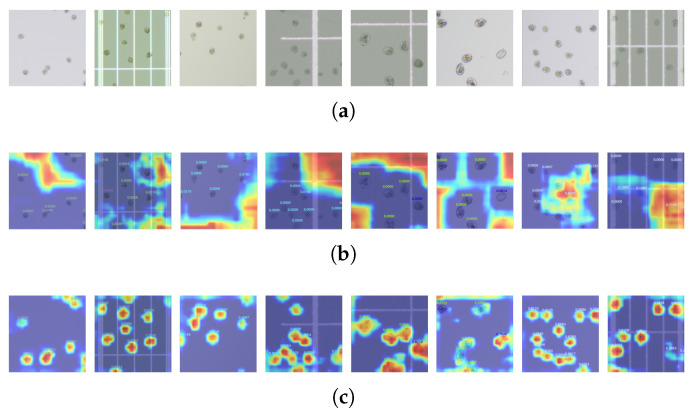
(**a**) Original image; (**b**) heatmap produced by the baseline network; (**c**) heatmap produced after introducing the EMA module.

To further verify the effectiveness of the EMA module, we generated heatmaps on the validation set for both the baseline network and the network incorporating the EMA module, as shown in Figure 15. The results show that after adding the EMA mechanism, the model can more effectively focus on the chicken coccidia oocyst, significantly reducing false detections and improving detection accuracy.

### 3.6. LFPD-Head Visualization

**Figure 16:**
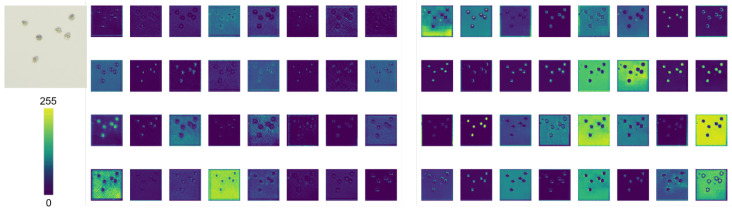
Left: Feature map without LFPD-Head output. Right: Feature map with LFPD-Head output.

To further verify the effectiveness of the LFPD-Head module, we visualized the feature maps of the head outputs from both the baseline network and the network with the LFPD-Head module on the validation set. As shown in Figure 16, after the introduction of LFPD-Head, the output feature map of the baseline model becomes richer, with clearer edge and texture information. This improvement reduces redundant features while strengthening and diversifying representative features, leading to a significant increase in detection accuracy.

### 3.7. Confusion Matrix Analysis

**Figure 17:**
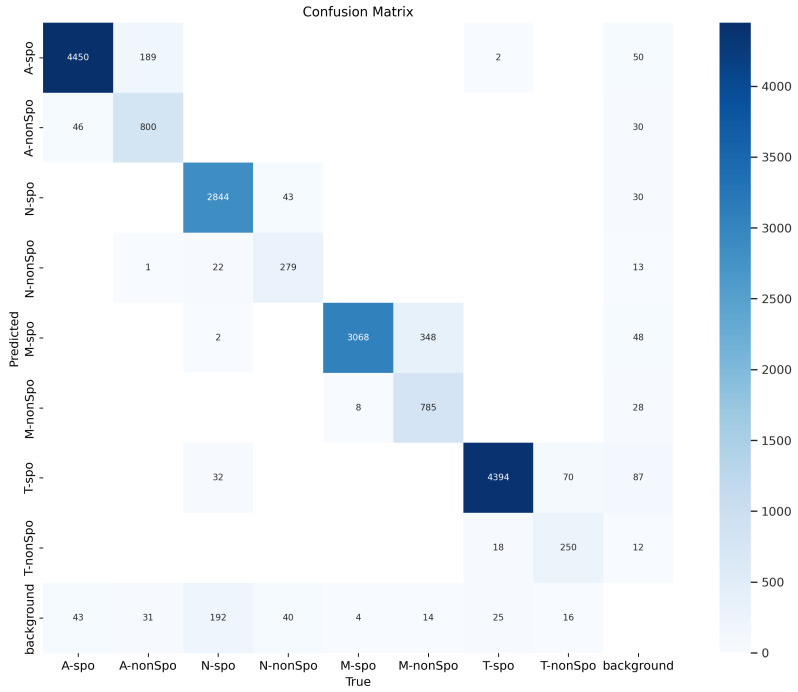
Confusion matrix on the test set.

Figure 17 shows the confusion matrix of the YOLO-Cocci model on the test set. As observed, the model’s recall for M-spo reached 99.6%, with almost no missed detections, indicating a strong perception of oocyst size and the ability to identify nearly all larger oocyst targets. For N-spo, the precision reached 97.6%, reflecting good detection accuracy. However, the model still misclassified 1% of the N-spo as T-spo, primarily due to the morphological similarity between the two types of oocysts, which resulted in a certain degree of confusion.

### 3.8. Visual Analysis of Detection Results

**Figure 18:**
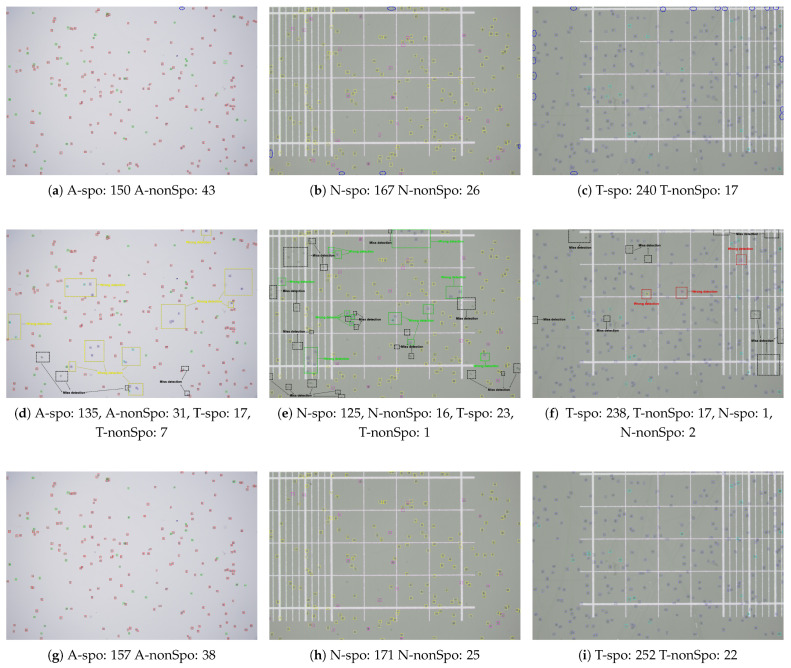
Visualization of detection results: (**a**–**c**) Manual annotation results; (**d**–**f**) detection results of the baseline network; (**g**–**i**) detection results of the YOLO-Cocci network.

For further validation of the advantages of the YOLO-Cocci model in chicken coccidia detection, several test images were randomly selected for visual comparison, as shown in Figure 18. As seen in the figure, the baseline network has many missed detections and false detections. This is primarily due to the small size of chicken coccidia oocysts in the image, with detection relying on the detailed information from low-level features. However, the baseline network fails to extract sufficient low-level features in the neck and detection head, leading to a high number of missed detections. Additionally, since E. acervulina, E. necatrix, and E. tenella share highly similar morphologies, the baseline model is prone to false detections. Specifically, as shown in Figure 18d, the baseline model misidentifies 11.3% of A-spo as T-spo; Figure 18e shows that 13.8% of N-spo are misidentified as T-spo; and Figure 18f shows that the baseline model misidentifies one T-spo as N-spo and two T-nonSpo as N-nonSpo. Moreover, the baseline model exhibits a significant number of missed detections (as shown in the black dotted box in the figure). In contrast, the YOLO-Cocci model achieves significant improvements in reducing missed detections and false detections. For example, as shown in Figure 18g, the YOLO-Cocci model does not misidentify A-spo as T-spo; and in Figure 18h, it does not misidentify N-spo as T-spo, showing its great potential in identifying morphologically similar chicken coccidia. Furthermore, the YOLO-Cocci model correctly detects chicken coccidia targets that were artificially missed at the edge of the image (as shown by the blue dotted ellipse in the figure), demonstrating its exceptional robustness. In summary, the proposed model has obvious advantages in chicken coccidia detection, significantly reducing missed and false detections, thus providing reliable technical support for chicken coccidia detection in vaccine environments.

### 3.9. Deployment and Application of YOLO-Cocci Model

**Figure 19:**
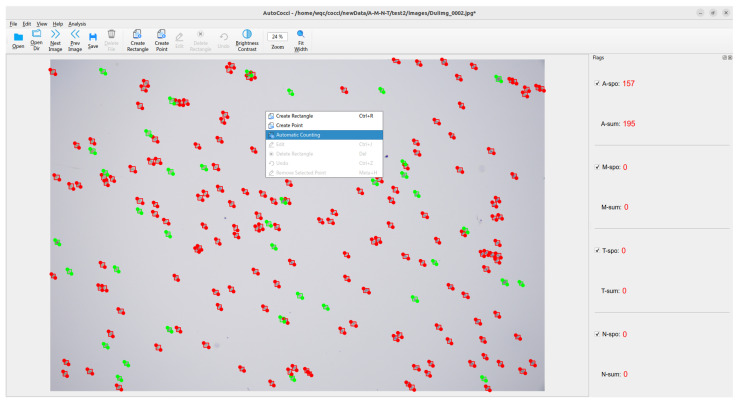
AutoCocci main interface. Red boxes indicate A-spo, while green boxes indicate A-nonSpo.

To verify the application potential of the improved model and effectively support automated detection in vaccine environments, we deployed the model as an algorithm server, called via the self-developed interactive client software AutoCocci, for automatic detection and result visualization. Users can click the “Automatic Counting” button in AutoCocci, which will automatically invoke the improved detection algorithm. The results are then returned to AutoCocci and displayed. The specific visualization interface is shown in Figure 19.

In actual deployment, the YOLO-Cocci model runs on a server equipped with a GeForce RTX 3090 graphics card, and users can access the service via the AutoCocci client on their personal computers. Test results indicate that, for a single image with a resolution of 5440 × 3648, the average processing time is 3.5 s, of which the model processing takes only 0.42 s, while the remaining time is primarily spent on network transmission and client-side rendering of the results.

After automatic detection, if there are false detections of chicken coccidia oocysts, users can directly make fine adjustments to correct the results. This method enables farmers who may lack professional identification experience to inspect the quality of purchased vaccines, determine whether they meet standards before vaccinating chickens, prevent the adverse effects of substandard vaccines on poultry health, and ultimately promote animal welfare.

## 4. Discussion

The research goal of this paper was to achieve accurate detection of chicken coccidia oocysts in vaccine environments. To this end, we proposed a chicken coccidia detection model, YOLO-Cocci, which is based on YOLOv8n and optimized in the backbone, neck, and detection head. These optimizations significantly improved the model’s performance in mAP@0.5 and mAP@0.5:0.95, while effectively reducing model parameters and computational burden. YOLO-Cocci enhances the ability to differentiate morphologically similar chicken coccidia. To support the automated detection of chicken coccidia in vaccine environments, we also developed a user-friendly interactive software, AutoCocci.

Despite marked enhancements demonstrated by the proposed model on various evaluation metrics, the experimental results show that the YOLO-Cocci model still exhibits missed detections in some cases, resulting in the recall rate not reaching an ideal level. In addition, false detections could result in higher-than-expected concentrations of sporulated oocysts in the produced vaccine, reducing its efficacy and ultimately increasing disease risk in vaccinated chickens.

Due to environmental noise—such as lighting variations and insufficient focus during image acquisition—as well as sample variability, these factors may negatively affect detection accuracy. The dataset used in this study was obtained from Foshan Standard Biotechnology Co., Ltd., ensuring consistency in data quality but potentially introducing bias in cross-institutional validation. To overcome these limitations, future research can explore enhanced image acquisition and preprocessing methods, such as denoising and contrast enhancement techniques, combined with more diverse training data and data augmentation strategies, to further improve the model’s robustness and generalization ability. Furthermore, future research will also conduct cross-institutional evaluation and integrate data from different sources to further validate and enhance the generalizability of the proposed method. Moreover, this detection task has inherent limitations: because some chicken coccidia oocysts are difficult to identify in images, manual labeling may involve uncertainties, leading to potential mislabeling.

During the model’s practical deployment, feedback from technical experts indicated that despite strong detection accuracy, certain challenges remain in real-world applications. For example, the model may still misidentify morphologically similar chicken coccidia. Based on this valuable feedback, we plan to further enhance the model’s feature extraction capabilities and introduce additional data enhancement techniques, such as using generative adversarial networks to enhance and balance samples, in order to improve the model’s detection accuracy further.

The EMA and IMFPN code, along with the 83 test images used in this study, are freely available at https://pan.baidu.com/wqc/bubua12 (accessed on 24 August 2025).

In future work, we plan to expand the detection model to cover more categories of chicken coccidia and further enhance its detection accuracy. To achieve this, we will further explore efficient methods for feature extraction and object detection. Additionally, we will explore real-time detection and lightweight technologies for chicken coccidia, aiming to integrate the detection algorithm into embedded devices, such as microscopes, to meet the clinical needs for chicken coccidiosis detection.

## 5. Conclusions

This paper proposes a method for detecting chicken coccidia oocysts in vaccine environments. To improve detection accuracy, we introduced the EMA, IMFPN, and LFPD-Head modules into YOLOv8n and developed YOLO-Cocci. Experimental results on a custom chicken coccidia dataset indicate that YOLO-Cocci achieves 89.6% in mAP@0.5, which is 6.5% higher than the baseline model. In the Eimeria necatrix detection task, mAP@0.5 is increased by up to 14%, significantly enhancing the model’s ability to distinguish morphologically similar chicken coccidia. Additionally, the model’s parameters and FLOPs are reduced to 2.59M and 7.1G, respectively, and the overall performance is better than other advanced object detection models. We also developed an interactive client software to verify the feasibility of the improved detection algorithm and provide users with convenient functions for automatic detection and visualization of detection results. This method provides a stable and highly accurate solution for the automatic detection of chicken coccidia in vaccine environments, effectively reducing the burden of manual detection, improving detection efficiency, and thus promoting the improvement of animal welfare levels.

## Figures and Tables

**Table 1:** Number distribution of chicken coccidia images per category across different carriers.

Carrier	Acervulina	Necatrix	Maxima	Tenella
Glass slide	50	51	59	50
Counting chamber	50	56	52	52

**Table 2:** Model comparison study.

Model	mAP@0.5	mAP@0.5:0.95	P	R	Params	FLOPs	FPS
YOLOv5n	87.6	65.8	89.1	83.6	1.77 M	4.2 G	**41**
YOLOv9t	88.6	66.6	92.3	83.9	2.60 M	10.7 G	19
YOLOv10n	89.5	66.1	91.1	**86.4**	2.70 M	8.2 G	15
YOLOv11n	88.3	66.6	92.8	84.0	2.58 M	6.3 G	16
RetinaNet	60.0	40.7	92.7	60.0	56.86 M	295 G	3
Faster R-CNN	73.5	48.8	85.4	76.5	60.64 M	265 G	4
Mask R-CNN	74.7	49.2	86.1	75.8	62.28 M	265 G	2
**YOLO-Cocci (ours)**	**89.6**	**67.3**	**92.9**	86.0	2.59 M	7.1 G	17

**Bold** indicates the best result.

**Table 3:** Multi-scale kernel design.

Kernel Design	Params	FLOPs	mAP@0.5
(3, 3, 3, 3)	2.48 M	6.7 G	87.0
(3, 5, 7, 9)	2.54 M	6.9 G	88.6
(5, 7, 9, 11)	2.59 M	7.1 G	**89.6**
(7, 9, 11, 13)	2.66 M	7.3 G	89.0
(3, 7, 11, 15)	2.65 M	7.3 G	88.0
(3, 5, 7, 9, 11)	2.60 M	7.1 G	87.8

**Bold** indicates the best result.

**Table 4:** Ablation study.

Model	mAP@0.5	mAP@0.5:0.95	A-spo	A-nonSpo	N-spo	N-nonSpo	M-spo	M-nonSpo	T-spo	T-nonSpo	Params	FLOPs
Baseline	83.1	62.3	88.6	80.9	80.9	71.4	90.3	83.0	94.1	75.7	3.01 M	8.1 G
+IMFPN	86.8	65.1	93.6	85.6	87.6	78.9	91.2	82.8	95.2	79.4	3.04 M	9.4 G
+EMA	88.4	66.3	94.4	85.3	91.9	80.5	90.7	**83.4**	**97.9**	83.3	3.02 M	8.2 G
+LFPD-Head	87.6	65.3	93.5	85.3	89.6	79.0	90.1	83.1	97.4	82.5	2.53 M	5.7 G
+IMFPN+EMA	89.1	67.1	94.7	86.2	93.9	82.1	**91.4**	83.2	97.5	83.9	3.05 M	9.5 G
+IMFPN+LFPD-Head	88.7	66.7	95.3	86.0	93.8	80.2	90.7	82.7	97.9	82.9	2.58 M	7.0 G
+EMA+LFPD-Head	89.0	66.3	95.0	86.1	**95.7**	82.8	89.8	82.5	97.3	82.4	2.54 M	5.7 G
**YOLO-Cocci (ours)**	**89.6**	**67.3**	**95.6**	**86.6**	94.9	**83.9**	90.6	83.2	97.7	**84.0**	2.59 M	7.1 G

**Bold** indicates the best result.

## Data Availability

The datasets presented in this article are not readily available as they are part of an ongoing research project.

## Abbreviations

The following abbreviations are used in this manuscript:

YOLO	You Only Look Once
EMA	Efficient multi-scale attention
IMFPN	Inception-style multi-scale fusion pyramid network
LFPD	Lightweight feature-reconstructed and partially decoupled

## References

[1] Mesa-Pineda C., Navarro-Ruíz J.L., López-Osorio S., Chaparro-Gutiérrez J.J., Gómez-Osorio L.M. (2021). Chicken coccidiosis: From the parasite lifecycle to control of the disease. Front. Vet. Sci..

[2] Blake D.P., Tomley F.M. (2014). Securing poultry production from the ever-present Eimeria challenge. Trends Parasitol..

[3] Fatoba A.J., Adeleke M.A. (2018). Diagnosis and control of chicken coccidiosis: A recent update. J. Parasit. Dis..

[4] Abebe E., Gugsa G. (2018). A review on poultry coccidiosis. Abyssinia J. Sci. Technol..

[5] Hamid P.H., Kristianingrum Y.P., Wardhana A.H., Prastowo S., Silva L.M.R.d. (2018). Chicken coccidiosis in Central Java, Indonesia: A recent update. Vet. Med. Int..

[6] Blake D.P., Knox J., Dehaeck B., Huntington B., Rathinam T., Ravipati V., Ayoade S., Gilbert W., Adebambo A.O., Jatau I.D. (2020). Re-calculating the cost of coccidiosis in chickens. Vet. Res..

[7] Peek H., Landman W. (2011). Coccidiosis in poultry: Anticoccidial products, vaccines and other prevention strategies. Vet. Q..

[8] Haug A., Williams R., Larsen S. (2006). Counting coccidial oocysts in chicken faeces: A comparative study of a standard McMaster technique and a new rapid method. Vet. Parasitol..

[9] Jarquín-Díaz V.H., Balard A., Ferreira S.C.M., Mittné V., Murata J.M., Heitlinger E. (2022). DNA-based quantification and counting of transmission stages provides different but complementary parasite load estimates: An example from rodent coccidia (Eimeria). Parasites Vectors.

[10] Ahmed-Laloui H., Zaak H., Rahmani A., Dems M.A., Cherb N. (2022). A Simple Spectrophotometric Method for Coccidian Oocysts Counting in Broiler Feces. Acta Parasitol..

[11] Adams D.S., Kulkarni R.R., Mohammed J.P., Crespo R. (2022). A flow cytometric method for enumeration and speciation of coccidia affecting broiler chickens. Vet. Parasitol..

[12] Boyett T., Crespo R., Vinueza V.C., Gaghan C., Mohammed J.P., Kulkarni R.R. (2022). Enumeration and speciation of coccidia affecting turkeys using flow cytometry method. J. Appl. Poult. Res..

[13] Adams D.S., Ruiz-Jimenez F., Fletcher O.J., Gall S., Crespo R. (2022). Image analysis for Eimeria oocyst counts and classification. J. Appl. Poult. Res..

[14] Viet N.Q., ThanhTuyen D.T., Hoang T.H. Parasite worm egg automatic detection in microscopy stool image based on Faster R-CNN. Proceedings of the 3rd International Conference on Machine Learning and Soft Computing.

[15] Tahir M.W., Zaidi N.A., Rao A.A., Blank R., Vellekoop M.J., Lang W. (2018). A fungus spores dataset and a convolutional neural network based approach for fungus detection. IEEE Trans. Nanobiosci..

[16] Panicker R.O., Kalmady K.S., Rajan J., Sabu M. (2018). Automatic detection of tuberculosis bacilli from microscopic sputum smear images using deep learning methods. Biocybern. Biomed. Eng..

[17] Devi P., Subburamu K., Giridhari V.A., Dananjeyan B., Maruthamuthu T. (2025). Integration of AI based tools in dairy quality control: Enhancing pathogen detection efficiency. J. Food Meas. Charact..

[18] Jafar A., Bibi N., Naqvi R.A., Sadeghi-Niaraki A., Jeong D. (2024). Revolutionizing agriculture with artificial intelligence: Plant disease detection methods, applications, and their limitations. Front. Plant Sci..

[19] Oon Y.L., Oon Y.S., Ayaz M., Deng M., Li L., Song K. (2023). Waterborne pathogens detection technologies: Advances, challenges, and future perspectives. Front. Microbiol..

[20] Zhou C., He H., Zhou H., Ge F., Yu P. (2025). MSRT-DETR: A novel RT-DETR model with multi-scale feature sequence for cell detection. Biomed. Signal Process. Control.

[21] Chen T., Chefd’Hotel C. (2014). Deep learning based automatic immune cell detection for immunohistochemistry images. International Workshop on Machine Learning in Medical Imaging.

[22] Moen E., Bannon D., Kudo T., Graf W., Covert M., Van Valen D. (2019). Deep learning for cellular image analysis. Nat. Methods.

[23] Abdurahman F., Fante K.A., Aliy M. (2021). Malaria parasite detection in thick blood smear microscopic images using modified YOLOV3 and YOLOV4 models. BMC Bioinform..

[24] Ren S., He K., Girshick R., Sun J. (2016). Faster R-CNN: Towards real-time object detection with region proposal networks. IEEE Trans. Pattern Anal. Mach. Intell..

[25] Liu W., Anguelov D., Erhan D., Szegedy C., Reed S., Fu C.Y., Berg A.C. (2016). Ssd: Single shot multibox detector. Computer Vision–ECCV 2016: 14th European Conference, Amsterdam, The Netherlands, 11–14 October 2016, Proceedings, Part I 14.

[26] Kumar S., Arif T., Ahamad G., Chaudhary A.A., Khan S., Ali M.A. (2023). An efficient and effective framework for intestinal parasite egg detection using YOLOv5. Diagnostics.

[27] Smith M.K., Buhr D.L., Dhlakama T.A., Dupraw D., Fitz-Coy S., Francisco A., Ganesan A., Hubbard S.A., Nederlof A., Newman L.J. (2023). Automated enumeration of Eimeria oocysts in feces for rapid coccidiosis monitoring. Poult. Sci..

[28] Kellogg I., Roberts D.L., Crespo R. (2024). Automated image analysis for detection of coccidia in poultry. Animals.

[29] He K., Gkioxari G., Dollár P., Girshick R. Mask r-cnn. Proceedings of the IEEE International Conference on Computer Vision.

[30] Bochkovskiy A., Wang C.Y., Liao H.Y.M. (2020). Yolov4: Optimal speed and accuracy of object detection. arXiv.

[31] Zhang H. (2017). mixup: Beyond empirical risk minimization. arXiv.

[32] Ouyang D., He S., Zhang G., Luo M., Guo H., Zhan J., Huang Z. Efficient multi-scale attention module with cross-spatial learning. Proceedings of the ICASSP 2023–2023 IEEE International Conference on Acoustics, Speech and Signal Processing (ICASSP).

[33] Hou Q., Zhou D., Feng J. Coordinate attention for efficient mobile network design. Proceedings of the IEEE/CVF Conference on Computer Vision and Pattern Recognition.

[34] Woo S., Park J., Lee J.Y., Kweon I.S. Cbam: Convolutional block attention module. Proceedings of the European Conference on Computer Vision (ECCV).

[35] Wu Y., He K. Group normalization. Proceedings of the European conference on computer vision (ECCV).

[36] Liu S., Qi L., Qin H., Shi J., Jia J. Path aggregation network for instance segmentation. Proceedings of the IEEE Conference on Computer Vision and Pattern Recognition.

[37] Lin T.Y., Dollár P., Girshick R., He K., Hariharan B., Belongie S. Feature pyramid networks for object detection. Proceedings of the IEEE Conference on Computer Vision and Pattern Recognition.

[38] Li J., Wen Y., He L. Scconv: Spatial and channel reconstruction convolution for feature redundancy. Proceedings of the IEEE/CVF Conference on Computer Vision and Pattern Recognition.

[39] Li X., Wang W., Hu X., Yang J. Selective kernel networks. Proceedings of the IEEE/CVF Conference on Computer Vision and Pattern Recognition.

[40] Wang C.Y., Yeh I.H., Mark Liao H.Y. (2024). Yolov9: Learning what you want to learn using programmable gradient information. European Conference on Computer Vision.

[41] Wang A., Chen H., Liu L., Chen K., Lin Z., Han J. (2024). Yolov10: Real-time end-to-end object detection. Adv. Neural Inf. Process. Syst..

[42] Jocher G. (2024). Yolov11. https://github.com/ultralytics/ultralytics.

[43] Lin T.Y., Goyal P., Girshick R., He K., Dollár P. Focal loss for dense object detection. Proceedings of the IEEE International Conference on Computer Vision.

